# Novel Apigenin Based Small Molecule that Targets Snake Venom Metalloproteases

**DOI:** 10.1371/journal.pone.0106364

**Published:** 2014-09-03

**Authors:** Venkatachalaiah Srinivasa, Mahalingam S. Sundaram, Sebastian Anusha, Mahadevappa Hemshekhar, Siddaiah Chandra Nayaka, Kempaiah Kemparaju, Basappa, Kesturu S. Girish, Kanchugarakoppal S. Rangappa

**Affiliations:** 1 Laboratory of Chemical Biology, Department of Chemistry, Bangalore University, Bangalore, India; 2 Department of Studies in Biochemistry, University of Mysore, Mysore, India; 3 Department of Studies in Biotechnology, University of Mysore, Mysore, India; 4 Department of Studies and Research in Biochemistry, Tumkur University, Tumkur, India; 5 Department of Studies in Chemistry, University of Mysore, Mysore, India; Georgia Regents University, United States of America

## Abstract

The classical antivenom therapy has appreciably reduced snakebite mortality rate and thus is the only savior drug available. Unfortunately, it considerably fails to shield the viper bite complications like hemorrhage, local tissue degradation and necrosis responsible for severe morbidity. Moreover, the therapy is also tagged with limitations including anaphylaxis, serum sickness and poor availability. Over the last decade, snake venom metalloproteases (SVMPs) are reported to be the primary component responsible for hemorrhage and tissue degradation at bitten site. Thus, antivenom inability to offset viper venom-induced local toxicity has been a basis for an insistent search for SVMP inhibitors. Here we report the inhibitory effect of compound 5d, an apigenin based molecule against SVMPs both *in silico* and *in vivo*. Several apigenin analogues are synthesized using multicomponent Ugi reactions. Among them, compound 5d effectively abrogated *Echis carinatus* (EC) venom-induced local hemorrhage, tissue necrosis and myotoxicity in a dose dependant fashion. The histopathological study further conferred effective inhibition of basement membrane degradation, and accumulation of inflammatory leucocytes at the site of EC venom inoculation. The compound also protected EC venom-induced fibrin and fibrinogen degradation. The molecular docking of compound 5d and bothropasin demonstrated the direct interaction of hydroxyl group of compound with Glu146 present in hydrophobic pocket of active site and does not chelate Zn^2+^. Hence, it is concluded that compound 5d could be a potent agent in viper bite management.

## Introduction

Snake envenomation is a neglected tropical disease affecting a large population residing in resource poor settings that are away from the primary health care centers [1], [2]. Most snakebite incidents in tropical countries are inflicted by vipers, among which *Echis carinatus* (EC) accounts for thousands of deaths and much more morbidity in Asia [3]–[5]. A maximum number of viper bite survivors suffer from permanent physical disabilities and psychological problems. EC envenomation causes remarkable local tissue damage including hemorrhage, myonecrosis, edema, and blistering along with systemic effects such as systemic hemorrhage of vital organs, hormonal imbalance, altered hemostasis, renal malfunction and hypotension [6], [7]. These pathological disorders comprise a cascade of events attributed to the combined action of extracellular matrix (ECM) degrading enzymes and target specific toxins/enzymes of EC venom [8].

Though the mortality rate due to snakebite is reduced markedly with the use of antivenoms, the therapy is tagged with limitations including anaphylaxis, serum sickness and poor availability [9]. Moreover, the major hurdle in the viper bite management is the incompetence of antivenom against debilitating local manifestations. A large amount of evidence exists reporting the persistent local tissue necrosis and damage at the bitten region even after the neutralization of systemic toxicity by classic antivenom therapy and has emerged as a post-medicated risk [10], [11]. The major components responsible for the notorious local tissue damage and systemic hemorrhage following viper bite are snake venom metalloproteases (SVMPs). These enzymatic toxins are generally denoted as “spreading factors” as they facilitate the easy diffusion of target specific toxins/enzymes into circulation by degrading the proteins of basement membrane and the connective tissues surrounding blood vessels [12], [13]. Thus, inhibition of SVMPs not only blocks the local toxicity, but also increases the survival time of the victim by reducing the dispersal of systemic toxins. Consequently, inhibition of SVMPs is reflected as a rate limiting step in viper bite management. Based on these facts, basic researchers and medical practitioners have considered SVMPs as the prime target to diminish the local tissue damage and systemic hemorrhage [14], [15].

In view of the frightening encumbrance of antivenoms, there is a need for designing new therapeutic molecules to neutralize the continued local tissue destruction and life threatening systemic complications. So far, several studies have reported the inhibition of SVMPs and its pathological effects by different chelating agents, synthetic and bioactive molecules including terpenoids, sterols, polyphenols and flavonoids [15]–[17]. These molecules show inhibition towards different class of SVMPs to a varied extent.

Apigenin belongs to flavone class of compounds and is known to inhibit several clinically important enzymes and cure pathological disorders. In the recent past, several studies reported the mitigation of matrix metalloproteinases (MMPs) expression by apigenin in target cells, which is induced by several agents such as carcinogens, ultraviolet A (UVA 320–400 nm), phorbol myristate acetate (PMA), interleukin-1 beta (IL-1β) and tumor necrosis factor-alpha (TNF-α) [18]–[21]. Further, inhibitory action of apigenin or apigenin structural analogues against cutaneous inflammation and infection-induced inflammation is also demonstrated [22], [23]. However, no study claims the SVMP inhibitory efficacy of apigenin or derivatives having apigenin nucleus.

The current study therefore focuses on derivatives with apigenin nucleus as potential inhibitors of SVMPs. To accomplish this challenge, we have utilized the multi-component reaction approach to synthesize the library of apigenin based small molecules to target SVMP-induced pathological effects in experimental animals. Additionally, *in silico* molecular interaction data between lead compound and SVMP is also demonstrated using the Accelrys Discovery Studio software [24].

## Materials and Methods

Synthesis and characterization of various apigenin structural analogues were provided as supplementary data (**Data S1** and **Table S1**)

### Chemicals

*Echis carinatus* venom (EC venom) was obtained from Irula Snake Catchers, Chennai, India. Gelatin (Type A from porcine skin), fibrinogen (from human plasma fraction I) were purchased from Sigma chemicals, St. Louis, USA. Lactate dehydrogenase (LDH) and Creatine phosphokinase (CPK) commercial kits were purchased from AGAPEE Diagnostics Ltd. Kerala, India. All other chemicals were of analytical grade purchased from Sisco Research Laboratories (SRL), Mumbai, India.

### Experimental animals

Adult Swiss Albino mice weighing 20–25 g were used for pharmacological studies. Animals were collected from University Central Animal Facility and housed under a controlled environment. All experiments were approved by the Intuitional Animal Ethical Committee (UOM/IAEC/06/2011), Department of Studies in Zoology, University of Mysore, Mysore, and were in accordance with the guidelines of the Committee for the Purpose of Control and Supervision of Experiments on Animals (CPCSEA). Besides, experiment involving healthy human blood plasma was approved by the Institutional Human Ethical Committee (IHEC-UOM No. 75/Ph.D/2012–13).

### Molecular Docking studies

The co-crystal structure of the SVMP with bound POL647, a pepditomimetic inhibitor of bothropasin was used for structure-based molecular docking studies [25]. The software InsightII from Accelrys was used to obtain a full set of tools for molecular modeling, which includes molecular graphics and forcefield-based simulations as reported previously [26]. The co-crystal structure of SVMP and POL647 was retrieved (PDBID: 3DSL). The molecular modeling was achieved with commercially available InsightII, Discovery Studio (DS) Version 2.5 software packages. Before performing CDOCKER protocol of DS, the 3D structure of SVMP was cleaned and the size and spatial orientation of the active site was identified by considering the tools such as selecting structure molecule as receptor and binding site or the sphere was made using the available programmes of the software. All of the calculations were performed using the CHARMM force field. Each energy-minimized final docking position of the individual apigenin structural analogues was evaluated using the interaction score function in the CDOCKER module of DS version 2.5. Based on the low CDOCKER energy value, compound 5d was selected for further studies.

### Hemorrhagic activity

Hemorrhagic activity was determined according to the method of Kondo et al. [27]. Different doses of compound 5d (1∶0, 1∶1, 1∶5 and 1∶10 ratio; venom: compound; w/w) were pre-incubated with EC venom [2 µg; minimum hemorrhagic dose (MHD), which is defined as the amount of venom that produces a hemorrhagic diameter of 10 mm] in a total volume of 50 µL PBS (10 mM, pH 7.4) at 37°C for 10 min. The pre-incubated samples were then injected intradermally into groups of mice (n = 5) independently and respective control groups were included. For independent injection experiment, mice (n = 5) were intradermally injected with 2 µg of EC venom first, which was preceded by compound 5d injection at the same site in the ratio 1∶10 (venom: compound; w/w) at different time intervals (0, 2.5, 5, 7.5 and 10 min). The compound 5d dose was selected based on the results obtained in pre-incubation study of venom with compound 5d (twice the concentration of complete inhibition of hemorrhage). After 3 h, the experimental mice were anaesthetized and the dorsal patch of skin was removed; the inner surface was observed for the hemorrhage and the diameter of the hemorrhagic spot was measured and photographed. Further, the skin tissues were processed for histopathology.

### Gelatinolytic activity

In order to determine the effect of compound 5d on gelatinolytic activity of EC venom, substrate gel assay was carried out as described by Heussen and Dowdle [28] with minor modifications. Briefly, gelatin was co-polymerized with a final concentration of 1% in to native-PAGE (10%). EC venom (3 µg) in the presence and absence of compound 5d was electrophoresed using basic-PAGE at 100 V at room temperature. The electrophoresed gel was incubated overnight at 37°C in 50 mM Tris-HCl buffer, pH 7.6 containing 150 mM NaCl, 10 mM CaCl_2_ and then stained with coomassie brilliant blue R-250. For inhibition studies, EC venom (3 µg) was pre-incubated independently with different doses of compound 5d (1∶0, 1∶1, 1∶2, 1∶5, 1∶10, 1∶25 and 1∶50 ratio; venom: compound; w/w) for 10 min at 37°C prior to electrophoresis. SVMP activity was detected as unstained translucent bands against dark blue back ground.

### Caseionolytic activity

To determine the effect of compound 5d on caseinolytic activity of EC venom, substrate gel assay was carried out as described by Nagaraju et al. [29]. Briefly, casein was co-polymerized with a final concentration of 0.2% in to native-PAGE (10%). EC venom (3 µg) in the presence and absence of compound 5d was electrophoresed using basic-PAGE at 100 V at room temperature. The electrophoresed gel was incubated overnight at 37°C in 50 mM Tris-HCl buffer, pH 7.6 containing 150 mM NaCl, 10 mM CaCl_2_ and then stained with coomassie brilliant blue R-250. For inhibition studies, EC venom (3 µg) was pre-incubated independently with different doses of compound 5d (1∶0, 1∶1, 1∶2, 1∶5, 1∶10, 1∶25 and 1∶50 ratio; venom: compound; w/w) for 10 min at 37°C prior to electrophoresis. Caseinolytic activity was detected as unstained translucent bands against dark blue back ground.

### UV-VIS Spectral Study

The interaction between cations such as Zn^2+^ and Ca^2+^ with compound 5d was studied by UV-VIS absorption spectroscopic scanning. Compound 5d (1 mM) was incubated with different concentrations of ZnCl_2_ and CaCl_2_ (0 to 2 mM) in 1 mL of PBS (10 mM, pH 7.4) and scanned with the wavelength range between 200–300 nm, using Beckman Coulter DU-730 spectrophotometer.

### Myotoxicity and myonecrosis

Effect of compound 5d on EC venom-induced myotoxicity was determined by following the method of Gutierrez et al. [30]. 5 µg of EC venom was pre-incubated with or without different doses of compound 5d (1∶5, 1∶10 and 1∶25; venom: compound; w/w) in a total reaction volume of 50 µL saline at 37°C for 10 min. Then the samples were injected intramuscularly into the right thigh of mice (n = 5). For independent injection experiment, mice (n = 5) were intramuscularly injected with EC venom later at different time intervals (0, 2.5, 5, 7.5 and 10 min) mice were injected with compound 5d at the ratio of 1∶50 (venom: compound; w/w) at the same site where venom was injected. The mice were anaesthetized and blood was collected by cardiac puncture. The thigh muscles were observed for damages, dissected and further processed for histopathology. Cytoplasmic marker enzymes lactate dehydrogenase (LDH) and creatine phosphokinase (CPK) were assessed in serum using AGAPEE diagnostic kits. Activities were expressed as unit/L.

### Edema inducing activity

The edema inducing activity was assessed according to the method of Yamakawa et al. [31]. EC venom [0.6 µg; minimum edema dose (MED), which is defined as the amount of protein required to cause an edema ratio of 120%] was pre-incubated with or without different doses of compound 5d (1∶1, 1∶5, 1∶10 and 1∶25 ratio; venom: compound; w/w) in a total reaction volume of 20 µL saline at 37°C for 10 min. Later on the samples were injected to groups of five mice in to the right footpads. The left footpad received saline, which served as control. Mice were sacrificed after 1 hr of sample injection and legs were dissected off at the ankle joint. An increase in weight due to edema was calculated as the edema ratio, which equals the weight of the edematous leg ×100/weight of control leg.

### Coagulant activity

The plasma coagulation property was determined according to the method of Condrea et al. [32]. Healthy human citrated plasma (200 µL) was incubated with 0.5 µg of EC venom and the clotting time was recorded after the addition of calcium chloride against a light source. Control tubes included citrated plasma incubated with PBS and calcium chloride or compound 5d alone. For inhibition studies, EC venom (0.5 µg) was pre-incubated with different doses of compound 5d (1∶1, 1∶5, 1∶10, 1∶25 and 1∶50 ratio; venom: compound; w/w).

### Fibrinogenolytic activity

Fibrinogenolytic activity was determined according to the method of Ouyang and Teng [33]. Human plasma fibrinogen (50 µg) was incubated with 0.2 µg of EC venom, which was pre-incubated with or without different doses of compound 5d (1∶1, 1∶5, 1∶10, 1∶25 and 1∶50 ratio; venom: compound; w/w) in 40 µL reaction volume of 10 mM Tris-HCl buffer pH 7.4, containing 10 mM NaCl at 37°C for 10 min. The reaction was terminated after 30 min by adding 20 µL denaturing buffer containing 1 M urea, 4% SDS and 4% β-mercaptoethanol. It was analyzed in 10% SDS-PAGE and the protein pattern was visualized by staining the gel with 0.1% coomassie brilliant blue R-250.

### Fibrinolytic activity

Fibrinolytic activity was determined using fibrin clot as a substrate. The fibrin clot was incubated with EC venom (0.2 µg) in 40 µL of 10 mM Tris-HCl buffer pH 7.4, containing 10 mM NaCl. The reaction was terminated after 30 min by adding 20 µL denaturing buffer containing 1 M urea, 4% SDS and 4% β-mercaptoethanol. An aliquot of 20 µL was analyzed in 10% SDS-PAGE and the protein pattern was visualized by staining the gel with 0.1% coomassie brilliant blue R-250. For inhibition studies, EC venom (0.2 µg) was pre incubated with different doses of compound 5d (1∶1, 1∶5, 1∶10, 1∶25 and 1∶50 ratio; venom: compound; w/w) at 37°C for 10 min.

### Histopathological studies

Skin and thigh muscle tissues were dissected out and fixed overnight in Bouin's solution. The tissue samples were dehydrated by treating with different grades of alcohol and chloroform: alcohol mixture. The processed tissue samples were embedded in molten paraffin wax, and 4 µm thick sections were prepared using microtome (Leica, Solms, Germany). The sections were stained with hematoxylin and eosin dye and were observed under Axio imager.A2 microscope and photographed.

### Protein estimation

Protein estimation was performed according to the method of Lowry et al. [34] using bovine serum albumin (BSA) as standard.

### Statistical analysis

Unless otherwise specified, the results are expressed as mean values ± SEM of five independent experiments. The data were compared by using analysis of variance (ANOVA) followed by the Tukey “honestly significantly different” (HSD) *post hoc* analysis. Significance was accepted for *p*<0.05 (*), *p*<0.01 (**) and *p*<0.001 (***). a - significant compared to saline control and b - significant compared to venom alone.

## Results

**Figure 1:**
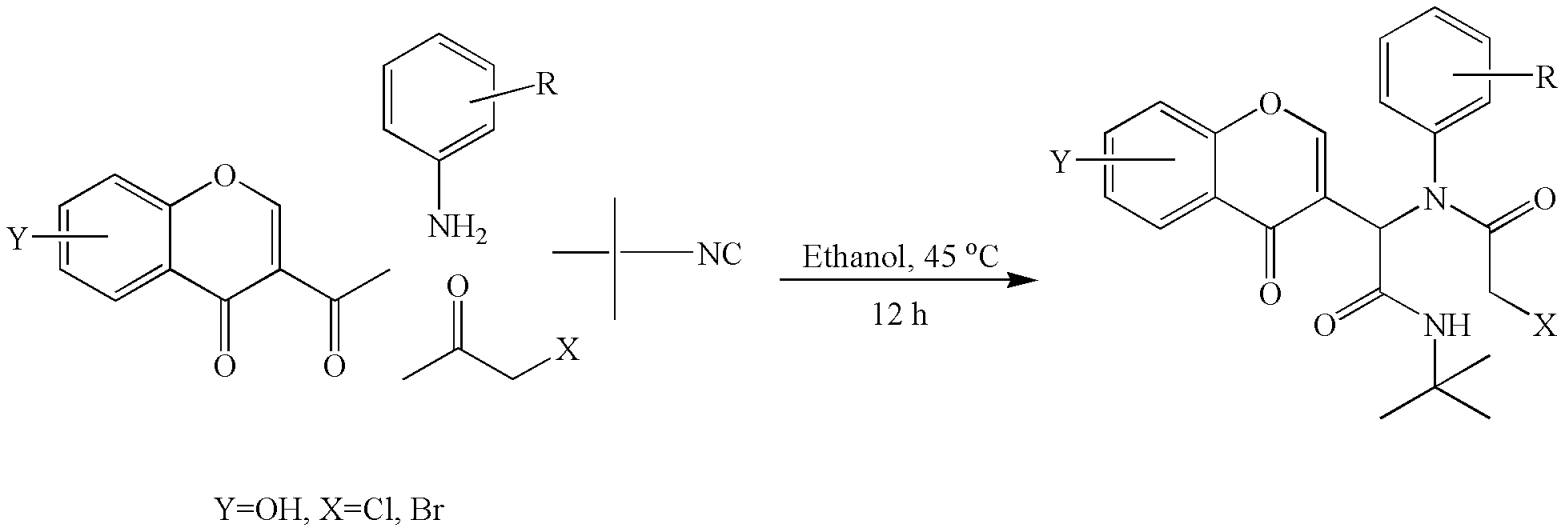
Schematic representation for the synthesis of apigenin structural analogues.

In the present study, we made an attempt to synthesize apigenin based small molecules with a flexible substitution at second position of the chroman moiety. A library of compounds was prepared by multi-component Ugi reaction using various aromatic amines, t-butyl-isocyanide and halo acetic acids (**Fig. 1**). The structures of the products were deduced based on IR, LC-MS, ^1^H NMR, and ^13^C NMR spectra (Table S1 and Data S1). During the preparation of the title compounds, the protocol was found to be effective with aromatic amines having electron donating groups and also, the *para*-methyl-substituted amines proceeded in shorter time. In contrast, the *ortho*-substituted amines took longer time to undergo products, which is likely due to its steric properties.

**Figure 2:**
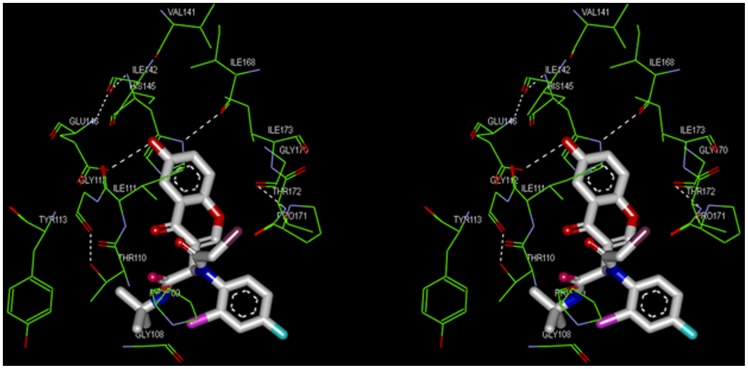
Stereo view of 3D structure of the SVMP (Bothropasin) showing molecular interaction with the compound 5d. The interaction between the compound and active site residues are shown using a line representation colored by parent atoms except for carbon (green color). The ligand is presented in the ball and stick form with their parent color except for carbon (white color). Hydrogen bonds are presented as white dotted lines.

Further, to examine the possible molecular interaction between SVMP and synthesized apigenin analogues, *in silico* molecular docking analysis were performed using Discovery Studio (DS) Version 2.5. The crystal structure of bothropasin, class III SVMP with the bound POL647 was used as a starting point. In order to know the molecular interaction between apigenin structural analogues and active site residues of bothropasin, molecular docking studies were performed. The results confirmed that the analogues showed high affinity towards active site residues with varied CDOCKER energy. Among the tested compounds, compound 5d was found to interact effectively with the active site of bothropasin with CDOCKER energy of 24.97 (**Table 1**). The flavone group of compound 5d was found buried into the active site grove *via* strong hydrogen bonding between the hydroxyl group and Glu146 of hydrophobic pocket that connects with Ile142 and His145 and other hydrophobic key amino acids, like val141, Ile168, Gly170, Pro171, Thr172, and Ile173. Additionally, acyl bromide tail of compound 5d formed hydrogen bonding with residues of active site including Thr110, Ile111, and Gly112 (**Fig. 2**). The acyl bromide tail also formed hydrophobic interaction with Gly108 and Pro109 residues. In addition, t-butyl group of amine of compound 5d was staked stacking with Tyr113 residue. In order to compare the results with a known SVMP inhibitors, POL647 and GM6001 were docked independently and ligands showed binding affinity with CDOCKER energy 22.45 and 0.37 respectively (**Table 1**). Based on the CDOCKER energy values, compound 5d was selected for further functional studies.

**Table 1:** Computational analysis of binding of compounds towards SVMP.

Compounds	-CDOCKER ENEGRY	-CDOCKER Interaction Energy
5a	30.8968	40.7197
5b	25.7389	46.1391
5c	30.1536	40.9792
5d	24.9736	39.1561
5e	28.55	40.201
5f	26.3275	42.4749
5g	28.6251	40.0069
5h	31.6015	44.2579
5i	32.4109	43.3109
5j	34.4272	45.0507
POL647	22.45	41.39
GM6001	0.37831	39.495

### Effect of compound 5d on EC venom-induced proteolytic activity

**Figure 3:**
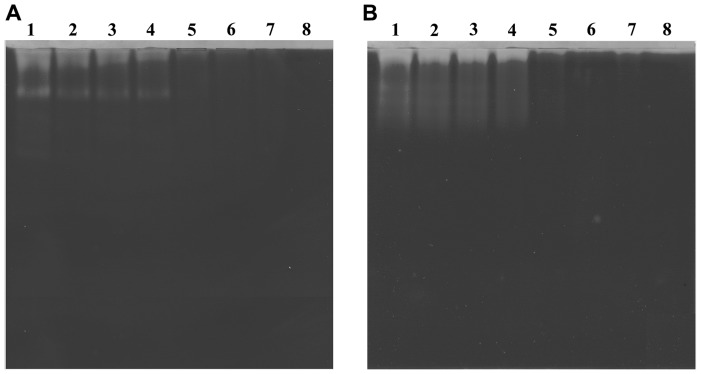
Effect of compound 5d on caseinolytic and gelatinolytic activities of EC venom. EC venom (3 µg) was pre-incubated separately with varied doses of compound 5d (1∶0; 1∶1; 1∶2; 1∶5; 1∶10; 1∶25 and 1∶50; venom: compound; w/w; Lane 1–7 respectively, Lane-8 represents 100 µg of compound 5d alone) for 10 min at 37°C. Samples were electrophoresed on gels impregnated separately with (A) casein (0.2%) and (B) gelatin (1%) as substrate. The gels were then processed as described in materials and methods section.

In order to evaluate the inhibitory efficacy of compound 5d on proteolytic activities of EC venom, zymography was carried out. **Figure 3A and 3B** represents the proteolytic activity of EC venom on casein and gelatin substrates respectively; the appearance of clear translucent bands against dark blue background suggests the caseinolytic and gelatinolytic activities. In both the cases, lane 1 represents the protease activity of EC venom in the absence of compound 5d. The results reveal that EC venom has SVMPs in a greater extent compared to serine proteases. Lane 2–7 represents the dose dependent inhibition of proteolytic activity by compound 5d. Complete inhibition was observed at the 1∶10 (venom: compound; w/w) ratio in both the cases. However, compound alone (200 µg) did not show any proteolytic activity on casein and gelatin.

### Effect of compound 5d on EC venom-induced hemorrhagic activity

**Figure 4:**
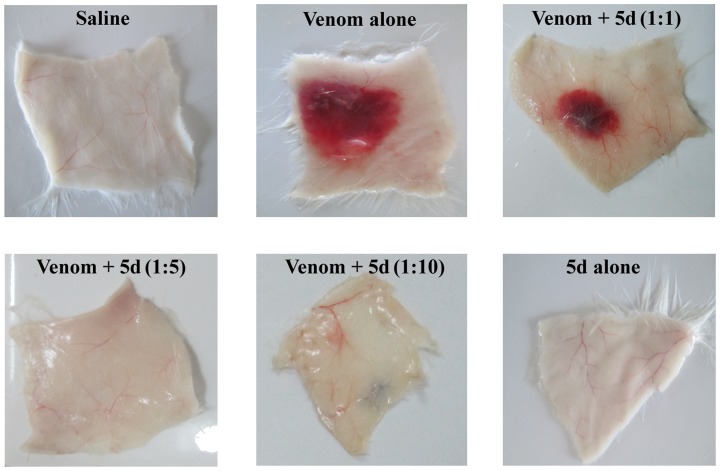
Neutralization of hemorrhagic activity of EC venom by compound 5d. EC venom (2 µg) was pre-incubated separately with various doses of compound 5d (1∶0 to 1∶10; venom: compound; w/w) in a total volume of 50 µL PBS for 10 min at 37°C. The samples were then injected intradermally into experimental animals (n = 5) and the assay was performed as described in materials and methods section. Results are expressed as repetitive pictures of three independent experiments.

The protective efficacy of compound 5d on EC venom-induced hemorrhage was assessed both in pre-incubation and independent injection experiments. Experimental mice were intradermally injected with either PBS or EC venom, and then the size of hemorrhage was measured 3 h post-injection. 2 µg of EC venom induced a hemorrhagic halo of 11 mm. In case of pre-incubation study, the EC venom-induced hemorrhage was dose dependently inhibited by compound 5d and complete inhibition was found at the ratio of 1∶5 (venom: compound; w/w) (**Fig. 4**). On the other hand, hemorrhage was significantly inhibited by compound (1∶10, w/w) when injected even after the time interval of 0, 2.5 and 5 min with a protection of 73%, 64% and 55% respectively in the independent study. However, inhibition was not significant beyond 7.5 min delay in compound 5d administration (**Table 2**). These results confirmed that pre-treatment with compound largely prevented hemorrhage induced by EC venom; however protection was found to be lesser extent in an independent injection experiment.

**Table 2:** Effect of compound 5d on EC venom-induced hemorrhage in an independent injection experiment.

Groups (n = 5)	Delay in compound 5d administration (min)	Diameter of hemorrhagic spot (mm) (mean ± SEM)	Inhibition (%)
Group I - Saline control	-	0	-
Group II - Venom alone	-	11±3	-
Group - III	0	3±1	73**
Group - IV	2.5	4±2	64**
Group - V	5.0	5±2	55**
Group - V	7.5	8±3	27*
Group - VII	10.0	10±2	9

EC venom (2 µg) was intradermally injected to the group of mice (n = 5) followed by injection of compound 5d (20 µg) at various time intervals (0, 2.5, 5, 7.5 and 10 min) to the same site where venom had been injected, and then the respective assay was performed as described in the materials and methods section. Data are presented as the mean ± SEM, ** *p*<0.01; * *p*<0.05.

**Figure 5:**
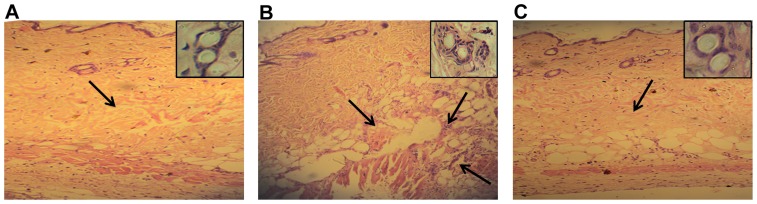
Light microphotograph of mice skin sections and blood vessels. EC venom (2 µg) was pre-incubated separately with compound 5d for 10 min at 37°C. The samples were injected intradermally into a groups of mice (n = 5) in a total volume of 50 µL saline. Mice were anaesthetized and sacrificed after 3 h, the dorsal patch of skin tissue was removed and the injected spot was processed for histopathological studies as described in materials and methods section. (A) Saline injected control section- note the intact ECM and the basement membrane surrounding the blood vessels. (B) EC venom-injected section shows the extensive disorganized dermis and epidermis layers. (C) EC venom pre-incubated with compound 5d (1∶5; venom: compound; w/w) injected section shows restoration of normal basement membrane. Original magnification 40x. The inset shows high power view of damaged and intact blood vessels.

So as to further delineate the microscopic alteration in dermis and epidermis of skin tissues, EC venom in presence or absence of compound injected spots were dissected out and processed for histological examination (**Fig. 5**). PBS injected skin possessed an intact basement membrane with no infiltration of inflammatory leucocytes (**Fig. 5A**). The EC venom-injected skin sections revealed an extensive degradation of ECM and basement membrane surrounding the blood vessels along with infiltration of inflammatory leucocytes (**Fig. 5B**). Pre-incubation of compound 5d with venom did not show any dermonecrosis and abolished the basement membrane degrading property of EC venom (**Fig. 5C**). EC venom-induced blood vessels damage and its restoration were ascertained by high resolution view of corresponding skin sections (**Fig. 5** **insets**). PBS injected skin revealed intact blood vessels with no infiltration of inflammatory leucocytes (**Fig. 5A** **inset**). In contrast, venom injected skin revealed damaged blood vessels along with the infiltration of inflammatory leucocytes (**Fig. 5B** **inset**). However, pre-incubation of venom with compound abrogated the blood vessels degrading property of EC venom (**Fig. 5C** **inset**).

### Effect of compound 5d on EC venom-induced myonecrosis and myotoxicity

**Figure 6:**
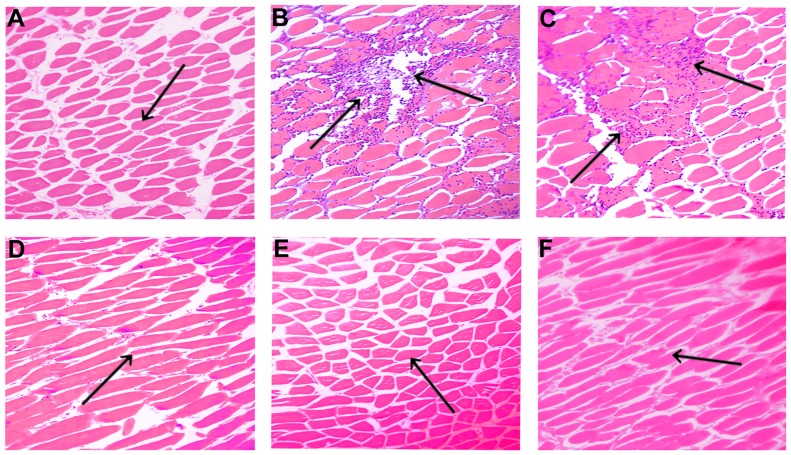
Light microphotograph of mice skeletal muscle sections. EC venom (5 µg) was pre-incubated separately with various doses of compound 5d (1∶0; 1∶5; 1∶10 and 1∶25; venom: compound; w/w) for 10 min at 37°C. The samples were injected intramuscularly into groups of mice (n = 5) in a total volume of 50 µL saline. Mice were anaesthetized and sacrificed after 3 h, the muscle tissue from the injected site was removed and processed for histopathological studies as described in materials and methods section. (A) Saline injected control section- note the intact basement membrane with striated myofibrils (B) EC venom-injected section shows the extensive disorganized myofibroblasts with infiltration of inflammatory leucocytes. (C)–(E) EC venom pre-incubated with the compound 5d injected section shows the inhibition and restoration of normal histology of muscle tissue (with respective doses of 1∶5; 1∶10 and 1∶25; venom: compound; w/w). (F) 150 µg Compound 5d alone injected section similar to saline control. Original magnification 40x.

To further examine the protective efficacy of compound 5d on EC venom-induced myonecrosis, both pre-incubation and independent injection experiments were carried out. Experimental mice were intramuscularly injected with either saline or EC venom, and then the muscle tissue from the injection site was dissected out 3 h post-injection. Saline injected muscle tissue showed intact myofibrils with myocytes (**Fig. 6A**). EC venom (5 µg) caused extensive myonecrosis of thigh muscle at the site of injection. The histopathological observations of muscle tissue sections injected with EC venom showed degraded and disordered myofibrils along with ruptured myocytes (**Fig. 6B**). Pre-incubation of EC venom with compound 5d effectively abrogated the muscle degradation dose-dependently, and complete restoration of muscle architecture was observed at 1∶25 (venom: compound; w/w) ratio (**Fig. 6D**).

**Figure 7:**
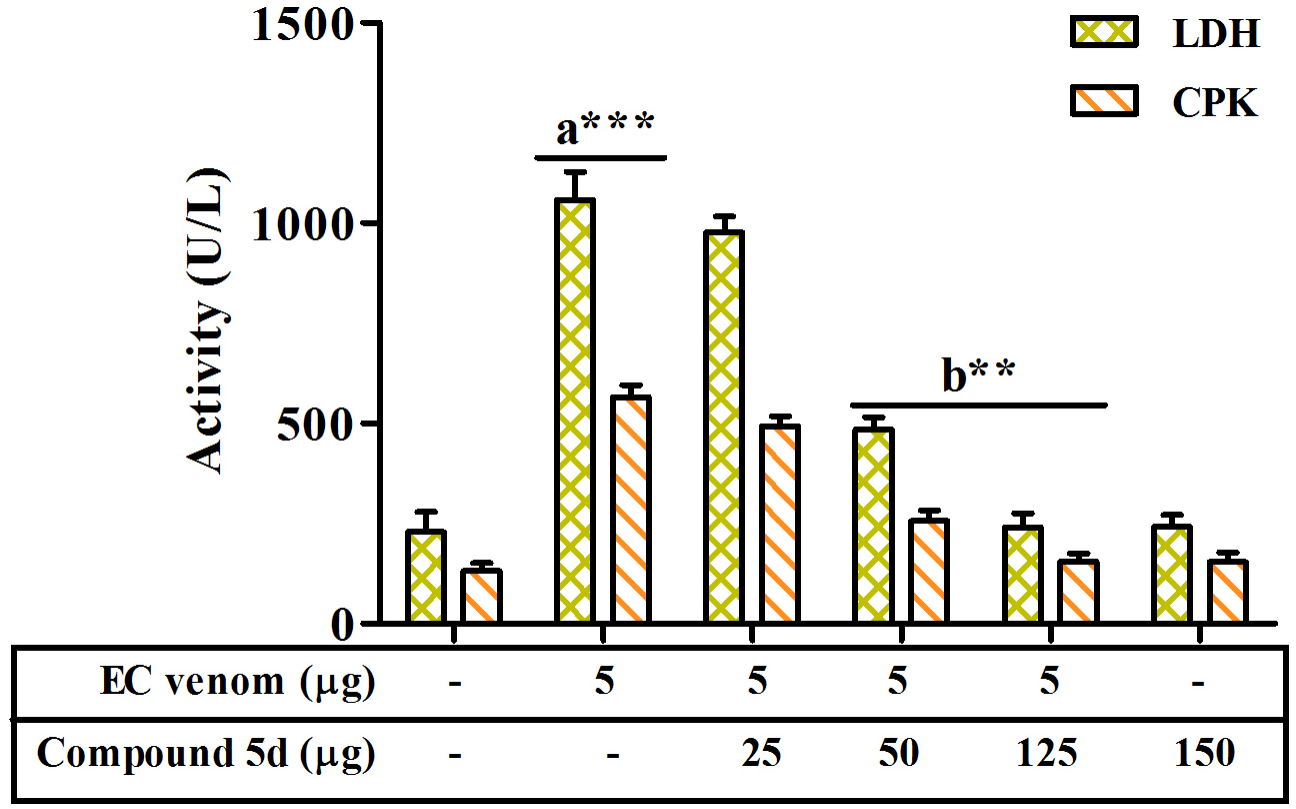
Effect of compound 5d on EC venom induced myotoxicity. EC venom (5 µg) was pre-incubated separately with varied doses of compound 5d (1∶0; 1∶5; 1∶10 and 1∶25; venom: compound; w/w) for 10 min at 37°C. The samples were injected intramuscularly into group of mice (n = 5) in a total volume of 50 µL saline. Mice were anaesthetized and sacrificed after 3 h and the assay was performed as described in materials and methods section. Results are expressed as mean ± SEM of three independent experiments. *** *p*<0.001, ** *p*<0.01, a - significant compared to saline control group and b - significant compared to venom alone group.

Elevated serum LDH and CPK activities further confirmed the EC venom-induced myotoxicity (**Fig. 7**). The compound 5d dose dependently abrogated the augmented LDH and CPK activities in pre-incubation experiment. On the other hand, independent injection of compound 5d at 0 and 2.5 min showed significant protection against myotoxicity but beyond 2.5 min it did not show any protection (**Table 3**).

**Table 3:** Effect of compound 5d on EC venom-induced serum LDH and CPK levels in experimental animals in an independent injection experiment.

Groups (n = 5)	Delay in compound 5d administration (min)	LDH (U/L) mean ± SEM	CPK (U/L) mean ± SEM
Group I - Saline control	-	230±16	132±12
Group II - Venom alone	-	1058±31^a^***	566±41^a^***
Group – III	0	312±20 ^b^***	174±26 ^b^***
Group – IV	2.5	523±23 ^b^*	246±21 ^b^*
Group – V	5.0	743±18	363±24
Group – VI	7.5	956±27	482±32
Group – VII	10.0	1032±28	554±37

EC venom (5 µg) was intramuscularly injected to the group of mice (n = 5) followed by compound 5d (250 µg) injection at various time intervals (0, 2.5, 5, 7.5 and 10 min) to the same site where venom had been injected, and then the respective assay was performed as described in the materials and methods section. Data are presented as the mean ± SEM, *** *p*<0.001, * *p*<0.05, a - significant compared to saline control group and b - significant compared to venom alone group.

### Effect of compound 5d on edema inducing activity of EC venom

Furthermore to probe the inhibitory efficacy of compound 5d against EC venom-induced edema, compound 5d treated EC venom was injected to foot pads of experimental animals. EC venom is well known to cause hemorrhagic edema, the extent of edema was found to be 142±5%. However, compound 5d failed to hinder the edema formation caused by EC venom. However, it abrogated the hemorrhage in the edematic paw in a dose dependent manner and complete inhibition was observed at 1∶5 ratio (venom: compound; w/w) (**Table 4**).

**Table 4:** Effect of compound 5d on EC venom-induced edema activity.

Groups (n = 5)	Percent edema ratio mean ±SEM	Visual rating for hemorrhage at right paw
Saline control	101±2	-
Venom alone (3MED, 0.6 µg)	142±5 ^a^***	+++
Venom: Compound 5d (1∶1, w/w)	138±4^‡^	++
Venom: Compound 5d (1∶5, w/w)	140±6^‡^	-
Venom: Compound 5d (1∶10, w/w)	139±6^‡^	-
Venom: Compound 5d (1∶25, w/w)	138±3^‡^	-
Compound 5d alone (15 µg)	101±3	-

EC venom (0.6 µg) was pre-incubated with compound 5d at various doses and injected to the right food pad to groups of mice (n = 5) and then the respective assay was performed as described in the materials and methods section. Data are presented as the mean ± SEM, *** *p*<0.001, a - significant compared to saline control group and ‡ - non-significant compared to venom alone group. The hemorrhage score is as follows: - nil; +++ High; ++ low.

### Effect of compound 5d on procoagulant activity of EC venom

In order to examine the inhibitory effect of compound 5d on EC venom-induced hemostasis alteration, recalcification time of citrated plasma was determined. 0.5 µg of EC venom exhibited potent procoagulant activity with clotting time of 45.0±8 sec, which is highly significant when compared to normal clotting time of 300±5 sec. Conversely, procoagulant activity of EC venom continued even with the increasing concentrations of compound 5d suggesting the inefficiency of compound 5d to inhibit the pro-coagulant nature of EC venom (**Table 5**).

**Table 5:** Effect of compound 5d on procoagulant activity of EC venom.

Groups (n = 5)	Plasma coagulation time (sec) mean ± SEM
CaCl_2_ alone	300±5
Venom alone (0.5 µg)	45±8^a^***
Venom: Compound 5d (1∶1, w/w)	44±6^‡^
Venom: Compound 5d (1∶5, w/w)	49±5^‡^
Venom: Compound 5d (1∶10, w/w)	47±4^‡^
Venom: Compound 5d (1∶25, w/w)	44±7^‡^
Venom: Compound 5d (1∶50, w/w)	47±6^‡^
Compound 5d alone (50 µg)	294±7

EC venom (0.6 µg) was pre-incubated with compound 5d at various doses and the procoagulant assay was performed as described in materials and methods section. Data are presented as the mean ± SEM, *** *p*<0.001, a - significant compared to saline control group and ‡ - non-significant compared to venom alone group.

### Effect of compound 5d on EC venom induced fibrin(ogen)olytic activity

**Figure 8:**
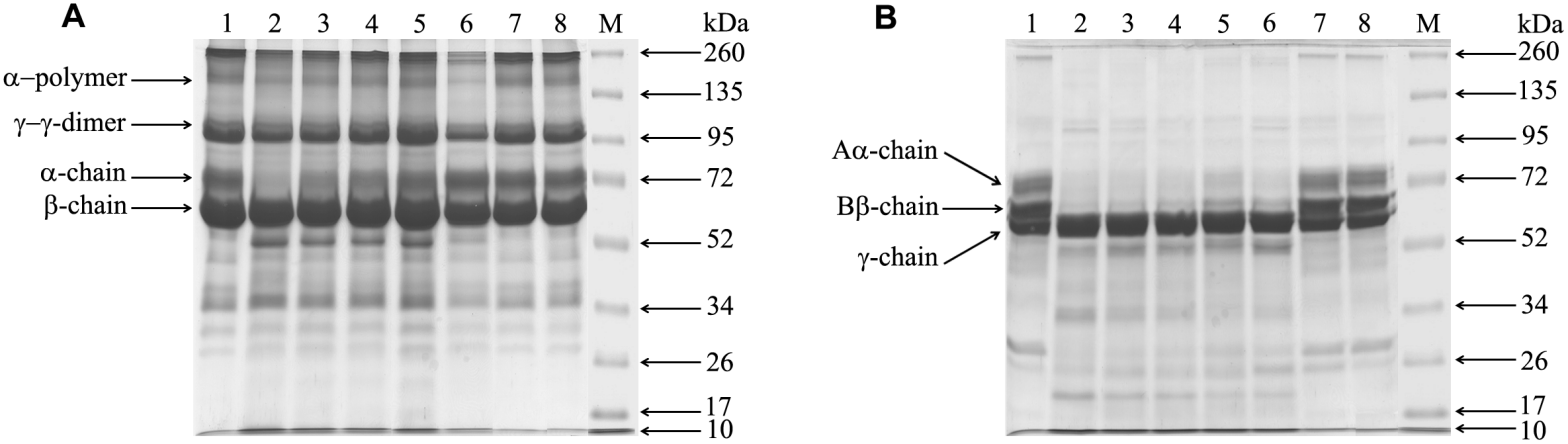
Effect of compound 5d on EC venom-induced fibrino(geno)lytic activity. EC venom (0.2 µg) was pre-incubated separately with different doses of compound 5d (1∶0; 1∶1; 1∶5; 1∶10; 1∶25 and 1∶50; venom: compound; w/w; Lane 2–7 respectively, Lane-1 represents substrate alone and Lane-8 represents substrate with 10 µg of compound 5d alone) for 10 min at 37°C. The reaction was initiated by adding respective substrates (A) fibrin and (B) fibrinogen and incubated for 30 min. Samples were electrophoresed on 10% SDS-PAGE and the gels were processed as described in materials and methods section.

Further the inhibitory effect of compound 5d on EC venom-induced fibrin(ogen)olytic activity was assessed, EC venom was incubated with fibrin or fibrinogen in presence or absence of compound 5d. EC venom (0.2 µg) specifically cleaved the α-polymer and α-chain of the fibrin molecule, in contrast γ-γ dimer and β-chain of the fibrin were found to be resistant to EC venom (**Fig. 8A**). The α-polymer and α-chain degradation property of EC venom on fibrin was dose dependently abrogated by compound 5d and complete inhibition was observed at 1∶25 (venom: compound; w/w) ratio. Further, EC venom specifically cleaved Aα chain and Bβ chain of fibrinogen but γ chain was resistant towards EC venom (**Fig. 8B**). The Aα and Bβ chain degradation property of EC venom was dose dependently abrogated by compound 5d and complete inhibition was observed at 1∶50 (venom: compound; w/w) ratio. These results confirmed that compound 5d largely prevented proteolytic activity of EC venom as suggested by inhibitory action against degradation of fibrin and fibrinogen.

### UV-Visible spectral study

**Figure 9:**
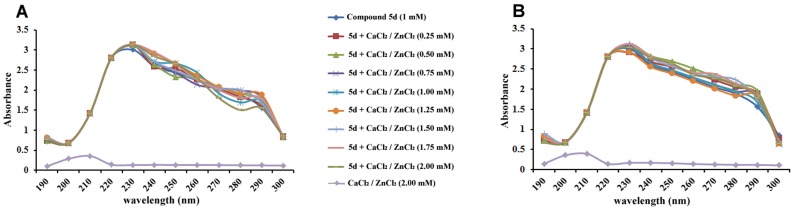
UV-VIS spectral studies of compound 5d in presence of CaCl_2_ and ZnCl_2_. The mixture of compound 5d (1 mM) and different concentrations of (A) CaCl_2_ and (B) ZnCl_2_ (0–2.0 mM) in a final volume of 1 mL PBS. The samples were monitored by spectroscopic scanning with the wavelength range of 200 to 300 nm.

In order to investigate the possible interaction between compound 5d and cations (Ca^2+^ and Zn ^2+^), UV-Vis spectra was monitored. The spectra of compound 5d (1 mM) with different concentration of CaCl_2_ and ZnCl_2_ (0–2 mM) were performed independently (**Fig. 9**). Compound 5d showed maximal absorption between 220 and 230 nm, the unaffected absorption spectra of compound 5d with increasing concentrations of CaCl_2_ (**Fig. 9A**) and ZnCl_2_ (**Fig. 9B**) revealed the absence of interaction between compound and either of the cations.

## Discussion

Snakebites still remain a neglected public health hazard and are common environmental and occupational disease throughout the world. Mortality rate due to snakebite is high in south Asia and particularly, in India the yearly estimates range from 1,364 to 50,000 [1]. Even though the medically approved antivenom therapy has drastically reduced the snakebite mortality rate, it does not protect viper venom-induced local tissue damage. In many instances, it has been a challenge to treat viper bite victims, which leads to permanent disabilities. The viper bite-mediated local toxicity is reported to be arbitrated by SVMPs [13], [15], [35]. Most of the viperid venoms are known to compose at least 32% SVMPs suggesting their potential role in viper bite pathology [36]. Thus, effective inhibition of these enzymes is a key in the viper bite management. The antivenom inability to offset viper venom-induced local toxicity has been the basis for an insistent search for SVMP inhibitors [37].

In the recent past, apigenin and its structural analogues are shown to inhibit MMPs in target cells induced by several agents such as carcinogens, ultraviolet A (UVA 320–400 nm), PMA, IL-1β and TNF-α. They are also demonstrated to be potent anti-cancer, anti-inflammatory and anti-bacterial agents [18]–[20]. Apigenin is known to mitigate a variety of anti-tumor effects, like gap junctional and intracellular communication stimulation along with inhibition of mutagenesis, transformation, angiogenesis and tumorigenesis [21]–[23]. With this background, apigenin structural analogues were synthesized using multi-component Ugi reaction. Presently, Ugi reaction has received much attention due to its simplicity and potentiality to synthesize desirable products with a good yield [38]. Ugi is an example of domino reaction, which has been used to convert a mixture of aldehyde, amine, carboxylic acid and isonitrile into complex structures providing linear peptide like adducts. These are important skeletal structures for the synthesis of a number of medicinal and biologically important compounds [39], [40]. With this concern, the current study probed the neutralizing efficacy of apigenin structural analogue (5d) on SVMP-induced pharmacological effects both in pre-incubation as well as independent injection experiments. In a similar study, we previously reported the PLA2 and cholinesterase inhibitory activities of novel isoxazolines and small oxazine compounds as anti-tumor agent [41]–[43].

Several studies have reported many potential synthetic SVMP inhibitors including Marimastat, CGS-270 23A, Bay-12 91566, AG-3340, etc. However, there exists a constant quest for a broad range of inhibitor effective against a range of SVMPs and crude snake venoms [13], [44]. Here, we demonstrate the ameliorative efficacy of compound 5d on EC venom-induced hemorrhage in a dose-dependent fashion suggesting its inhibitory action towards SVMPs. This was further supported by the histological examination. Further, gelatin and casein zymography confirmed dose-dependent abolition of the protease activity by compound 5d. Since a decade, studies have reported anti-hemorrhagic and anti-proteolytic efficacy of low molecular weight molecules (natural and synthetic), which generally act by chelation of Zn^2+^ and Ca^2+^cations. From the spectral results, it was very much clear that compound 5d does not chelate either Zn^2+^ or Ca^2+^ to inhibit SVMPs and serine proteases. However, it directly interacts with the active site Glu146 via strong hydrogen bond, which in turn connects with Ile142 and His145 and the other hydrophobic key amino acids Val141, Ile168, Gly170, Pro171, Thr172, and Ile173 as suggested by molecular docking of compound 5d with bothropasin. Having a non-chelating SVMP inhibitor is of great advantage as it offers no toxicity by chelating Ca^2+^ along with Zn^2+^ non-specifically as like EGTA, EDTA, TPEN, BAPTA, clodronate and doxycycline [5], [13], [44]. Moreover, compound 5d aligns at the top of this list as its efficacy falls in µM range similar to batimastat, whereas the rest mentioned are effective at mM range.

In addition, no effect on EC venom-induced procoagulant and edema inducing activities (Ca^2+^ dependent) by compound 5d suggests its inefficiency to chelate Ca^2+^ as evident by spectral studies. Ecarin, a class III procoagulant metalloprotease is mainly responsible for the pro-coagulant action of EC venom. Though P-III class SVMPs is capable of producing hemorrhage, ecarin is a non-hemorrhagic but a potent prothrombin activator [45]. Thus, it can be stated that compound 5d neither chelates Ca^2+^ nor inhibits ecarin. Nevertheless, compound 5d significantly attenuated the EC venom-induced fibrin and fibrinogen degradation, which might be by directly interacting with the enzyme and not by Ca^2+^ chelation. The ‘thrombin-like proteases’ present in EC venom interrupts the coagulation cascade and specifically hydrolyzes the fibrinogen by either releasing fibrinopeptide A or B or sometimes even both [46]. The fibrin monomers thus generated endure limited polymerization due to the loss of fibrinopeptide(s). Hence, fall short to activate factor VIII to VIIIa, which in turn fails to crosslink the fibrin monomers to form a hard clot. Consequently, compound 5d appears to be highly selective towards SVMPs and serine proteases. Furthermore, the compound 5d ameliorated the EC venom-induced myotoxicity and tissue necrosis. The histopathology of EC venom-injected longitudinal section of muscle tissue exhibited extensive necrosis, which was restored in the presence of compound 5d. This was further supported by the diminution of elevated serum levels of cytoplasmic markers, such as CPK and LDH activities by compound 5d to normal levels.

In conclusion, the current study undoubtedly revealed the abrogation of EC venom-induced local manifestations such as hemorrhage and persistent tissue necrosis by compound 5d, a derivative having apigenin nucleus. It was demonstrated that compound 5d selectively inhibits SVMPs both *in silico* and *in vivo*. The molecular docking studies conferred the direct interaction of compound 5d with P-III class SVMP bothropasin of *B. jararaca* at the catalytic site but it does not chelate either Zn^2+^ or Ca^2+^. Thus, compound 5d could be a primary agent in the management of viper venom-induced local tissue damage, which can reduce diffusion rate of systemic toxins in the snakebite victims. Hence, the current investigation demonstrates that the compound can be an effective auxiliary agent laterally with the existing antivenom therapy in the management of viper bites. Future studies related to protective efficacy of compound 5d against systemic hemorrhage of vital organs and possible viper venom-induced secondary complications including hormonal imbalance, renal malfunction, and infertility, are highly exciting. In addition, a high degree of structural and functional homology between SVMPs and MMPs suggests that compound 5d may find enormous value in the regulation of pathologies involving the participation of MMPs such as cancer, wound healing, inflammation and arthritis.

## Supporting Information

Table S1 **Various apigenin structural analogues synthesized as Ugi products.**
(DOCX)

Data S1 (DOCX)
