# Supplementary material for: Novel Apigenin Based Small Molecule that Targets Snake Venom Metalloproteases
Source: PLoS One. 2014 Sep 3;9(9):e106364. doi: 10.1371/journal.pone.0106364 (PMC4153592; doi:10.1371/journal.pone.0106364)
Supplement: Table S1 — Various apigenin structural analogues synthesized as Ugi products. (DOCX) [file pone.0106364.s001.docx]

**Novel Apigenin Structural Analogues that Target Snake Venom Metalloproteases**

Venkatachalaiah Srinivasa, Mahalingam S. Sundaram, Sebastian Anusha, Mahadevappa Hemshekhar, Siddaiah Chandra Nayaka, Kempaiah Kemparaju, Basappa, Kesturu S. Girish Kanchugarakoppal S. Rangappa

**Table S1.** Various apigenin structural analogues synthesized as Ugi products

| Reagents | | | | Product | Entry | Yield (%) | Time (hr) |
| --- | --- | --- | --- | --- | --- | --- | --- |
|  |  |  |  |  | 5a | 98 | 8 |
| 1a |  | 3a |  |  | 5b | 98 | 8 |
| 1a | 2b | 3a |  |  | 5c | 89 | 9 |
|  | 2b | 3a |  |  | 5d | 85 | 24 |
| 1a | 2a | 3a | 4d |  | 5e | 85 | 12 |
| 1a | 2a | 3a |  |  | 5f | 84 | 12 |
| 1a | 2b | 3a | 4c |  | 5g | 89 | 12 |
| 1a | 2a | 3a |  |  | 5h | 90 | 10 |
| 1a | 2b | 3a |  |  | 5i | 80 | 24 |
| 1a | 2b | 3a |  |  | 5j | 80 | 24 |
