# Supplementary material for: Novel Apigenin Based Small Molecule that Targets Snake Venom Metalloproteases
Source: PLoS One. 2014 Sep 3;9(9):e106364. doi: 10.1371/journal.pone.0106364 (PMC4153592; doi:10.1371/journal.pone.0106364)
Supplement: Data S1 — (DOCX) [file pone.0106364.s002.docx]

**Novel Apigenin Structural Analogues that Target Snake Venom Metalloproteases**

Venkatachalaiah Srinivasa, Mahalingam S. Sundaram, Sebastian Anusha, Mahadevappa Hemshekhar, Siddaiah Chandra Nayaka, Kempaiah Kemparaju, Basappa, Kesturu S. Girish Kanchugarakoppal S. Rangappa

**Data S1**

**General procedure for synthesis of various apigenin structural analogues**

4-oxo-4H-chromene-3-carbaldehyde (5.74 mM) and amine (5.74 mM) were dissolved in ethanol (10 mL) in a clean round bottom flask and stirred for 30 min at 45°C. To this reaction mixture, chloro/bromo acetic acid (5.74 mM) and t-butyl-isocyanide (5.74 mM) was added and the mixture was stirred continuously until the reaction gets completed. A Pale yellow colored solid product was separated out, filtered and washed with cold ethanol. The pure product was separated by column chromatography using neutral alumina.

**Synthesis of** **[(2-Chloroacetyl)(P-tolyl)amino]-2-(4-oxo-3-chromenyl)-1-(tert-butylamino) 1-ethanone (5a):** White solid, mp-194-196°C; ^1^H NMR( CDCl3, 400 MHz) δ: 1.366 (s, 9H- t-Butyl), 2.226 (s, 3H-Ar.Methyl,), 3.966 (s, 2H-methylene), 6.21 (s,1H-methine), 7.014 (s,1H =C-H), 6.374-6.398 (m,2H-Ar), 7.297-7.348 (m,2H-Ar), 7.573-7.973 (m,2H-Ar), 8.104-8.148 (m,2H-Ar); LCMS m/z: 441 [M+1]^+^;

**Synthesis of** **2-[(2-Bromoacetyl)(P-tolyl)amino]-2-(4-oxo-3-chromenyl)-1-(tert-butylamino)-1-ethanone (5b):** Yellow solid, mp-210-212°C; IR (υ_max_, cm-1): 3336, 3074, 1660, 1552: ^1^H NMR( CDCl3, 400 MHz) δ: 1.404 (s, 9H- t-Butyl), 2.293 (s, 3H-Ar.Methyl,), 3.873 (s, 2H-methylene), 6.376 (s,1H-methine), 7.146 (s,1H =C-H), 7.361-7.427 (m,4H-Ar), 7.632-7.674 (m,2H-Ar), 8.184-8.188 (m,2H-Ar); LCMS m/z: 485 [M+1] ^+;^, 486[M+2] ^+^;

**Synthesis of** **2-[(2-Bromoacetyl)(3,4-dimethoxyphenyl)amino]-2-(4-oxo-3-chromenyl)-1-(tert-butylamino)-1-ethanone (5c):** White solid, mp-220-221°C; ^1^H NMR( CDCl3, 400 MHz) δ: 1.304 (s, 9H- t-Butyl), 3.662 (s, 6H-Ar.O-Methoxy), 3.845 (s, 2H-methylene), 6.198 (s,1H-methine), 7.075 (s,1H =C-H), 6.527 (s, 1H-Ar), 6.27-6.527 (m,2H-Ar), 7.353-7.607 (m,2H-Ar), 7.999-8.134 (m,2H-Ar); LCMS m/z: 531[M+1] ^+^;

**Synthesis of** **2-[(2-bromoacetyl)(P-chlorophenyl)amino]-2-(4-oxo-3-chromenyl)-1-(tert-butylamino)-1-ethanone:- (5d):** Yellow solid, mp-178-183°C; ^1^H NMR( CDCl3, 400 MHz) δ: 1.337 (s, 9H- t-Butyl), 3.812 (s, 2H-methylene), 5.787 (s,1H-methine), 6.903 (s,1H =C-H), 6.753-7.004(m,2H), 6.600-(s,1H-Ar), 7.023-7.343 (m,4H-Ar), 7.37 (s,1H-Ar) ;LCMS m/z: 506.04 [[M+1] ^+^,

**Synthesis of** **2-[(2-Chloroacetyl)(P-chlorophenyl)amino]-2-(4-oxo-3-chromenyl)-1-(tert-butylamino)-1-ethanone (5e):** Pale yellow solid, mp-184-186°C; ^1^H NMR( CDCl3, 400 MHz) δ: 1.397 (s, 9H- t-Butyl), 3.645 (s, 2H-methylene), 6.207 (s,1H-methine), 7.401 (s,1H =C-H), 7.380-7.439 (m,2H-Ar), 7.653-7.692 (m,2H-Ar), 8.056-8.192 (m,4H-Ar); LCMS m/z: 506 [M+1];

**Synthesis of** **2-[(2-Chloroacetyl)(m-chlorophenyl)amino]-2-(4-oxo-3-chromenyl)-1-(tert-butylamino)-1-ethanone(5f):**  Yellow solid, mp-179-181°C; ^1^H NMR( CDCl3, 400 MHz) δ: 1.406 (s, 9H- t-Butyl), 3.650 (s, 2H-methylene), 6.217 (s,1H-methine), 7.010 (s,1H =C-H), 7.36-7.68(m,3H-Ar), 7.330 (s, 1H-Ar), 7.690-8.120 (m,4H-Ar); LCMS m/z: 506 [M+1], 507[M+2];

**Synthesis of** **2-[(2-Bromoacetyl)(3,4-dimethoxyphenyl)amino]-2-(7-hydroxy-4-oxo-3-chromenyl)-1-(tert-butylamino)-1-ethanone (5g):**  White solid, mp-191-193°C; ^1^H NMR( CDCl3, 400 MHz) δ: 1.344 (s, 9H- t-Butyl), 3.956 (s, 2H-methylene), 5.434 (brd,1H-OH), 5,899 (s,1H-methine) 7.063 (s,1H =C-H), 6.953-7.004(m,2H), 6600-(s,1H-Ar), 8.434-8.536 (m,2H-Ar), 7.831 (s,1H-Ar) ;LCMS m/z: 548.2 [M+1];

**Synthesis of** **2-[(2-Bromoacetyl)(P-hydroxyphenyl)amino]-2-(4-oxo-3-chromenyl)-1-(tert-butylamino)-1-ethanone (5h):** Pale yellow solid, mp-178-180°C; ^1^H NMR( CDCl3, 400 MHz) δ: 1.301 (s, 9H- t-Butyl), 3.618 (s, 2H-methylene), 6.226 (s,1H-methine), 5.36 (brd 1H-OH) 7.192 (s,1H =C-H), 7.294-7.592 (m,4H-Ar), 7.977-7.985 (m,2H-Ar), 8.123-8.143 (m,2H-Ar); LCMS m/z: 485 [M-2]^-^ ,486 [M-1];

**Synthesis of** **2-[(2-Bromoacetyl)(0-tolyl)amino]-2-(4-oxo-3-chromenyl)-1-(tert-butylamino)-1-ethanone (5i):**  Yellow solid, mp-210-212°C; ^1^H NMR( CDCl3, 400 MHz) δ: 1.414 (s, 9H- t-Butyl), 2.193 (s, 3H-Ar.Methyl,), 3.850 (s, 2H-methylene), 6.240 (s,1H-methine), 7.121 (s,1H =C-H), 7.342-7.457 (m,4H-Ar), 7.551-7.514 (m,2H-Ar), 8.164-8.168 (m,2H-Ar); LCMS m/z: 486 [M+1]^+^;

**Synthesis of** **2-[(2-Bromoacetyl)(o-chlorophenyl)amino]-2-(4-oxo-3-chromenyl)-1-(tert-butylamino)-1-ethanone (5j):**  Yellow solid, mp-172-174°C; ^1^H NMR( CDCl3, 400 MHz) δ: 1.403 (s, 9H- t-Butyl), 3.605 (s, 2H-methylene), 6.217 (s,1H-methine), 7.220 (s,1H =C-H), 7.19-7.439(m,4H-Ar), 7.650-7.658 (m, 2H-Ar), 8.046-8.190 (m,2H-Ar); LCMS m/z: 506 [M+1], 507[M+2];
